# Serum Cardiac and Skeletal Muscle Marker Changes in Repetitive Breath-hold Diving

**DOI:** 10.1186/s40798-021-00349-z

**Published:** 2021-08-21

**Authors:** Danilo Cialoni, Andrea Brizzolari, Nicola Sponsiello, Valentina Lancellotti, Cesare Lori, Gerardo Bosco, Alessandro Marroni, Alessandra Barassi

**Affiliations:** 1 Environmental Physiology and Medicine Laboratory, Department of Biomedical Sciences, University of Padua, Padua, Italy; 2 DAN Europe Research Division, Contrada Padune 11, 64026 Roseto degli Abruzzi, Italy; 3 Apnea Academy Research, Padua, Italy; 4 Cardiothoracic and Vascular Department, Azienda Ospedaliero-Universitaria Pisana (AOUP), Pisa, Italy; 5 Department of Health Science, Università degli Studi di Milano, Milan, Italy

**Keywords:** Breath-hold diving, Creatine kinase, Lactate dehydrogenase, Troponin, Physical activity

## Abstract

**Background:**

Breath-hold diving (BH-diving) is associated to extreme environmental conditions, prolonged physical activity, and complex adaptation mechanisms to supply enough O_2_ to vital organs. Consequently, one of the biggest effects could be an increased exercise-induced muscle fatigue, in both skeletal and cardiac muscles that can induce an increase of muscles injury markers including creatine kinase (CK), aspartate transferase (AST), and alanine transferase (ALT) when concerning the skeletal muscle, cardiac creatine kinase isoenzyme (CK-MBm) and cardiac troponin I (cTnI) when concerning the cardiac muscle, and lactate dehydrogenase (LDH) as index of muscle stress. The aim of this study is to investigate serum cardiac and skeletal muscle markers before and after a BH-diving training session.

**Results:**

We found statistically significant increases of CK (T0: 136.1% *p* < 0.0001; T1: 138.5%, *p* < 0.0001), CK-MBm (T0: 145.1%, *p* < 0.0001; T1: 153.2%, *p* < 0.0001) LDH (T0: 110.4%, *p* < 0.0003; T1: 110.1%, *p* < 0.0013) in both T0 and T1 blood samples, as compared to basal value. AST showed a statistically significant increase only at T0 (106.8%, *p* < 0.0007) while ALT did not exhibit statistically significant changes. We did not find any changes in cTnI levels between pre-dive and post-dive samples.

**Conclusions:**

Our data seem to indicate that during a BH-diving training session, skeletal and cardiac muscles react to physical effort releasing stress-related substances. Although the peculiar nature of BH-diving makes it difficult to understand if our results are related only to exercise induced muscle adaptation or whether acute hypoxia or a response to environmental changes (pressure) play a role to explain the observed changes, further studies are needed to better understand if these biomarker changes are linked to physical exercise or to acute hypoxia, or if both conditions play a role.

**Supplementary Information:**

The online version contains supplementary material available at 10.1186/s40798-021-00349-z.

## Key Points

- A single breath-hold diving (BH-diving) session can change some serum skeletal muscles injury markers including creatine kinase (CK), aspartate transferase (AST) and alanine transferase (ALT), and lactate dehydrogenase (LDH) as index of muscle stress.
- The physical effort related to the breath-hold diving session did not change cardiac troponin I (cTnI) while increases of cardiac creatine kinase isoenzyme (CK-MBm) were found.
- The peculiar nature of BH-diving makes it difficult to understand if the increases of muscle injury markers are related only to exercise or if both acute hypoxia and environmental changes (i.e., pressure) play a role to explain the observed changes.

## Introduction

Breath-hold diving (BH-diving) is the oldest underwater activity practiced for commercial, military purposes [1], and recently as a competitive sport [2]. BH-divers are exposed to extreme environmental conditions such as increased hyperbaric pressure and low temperature that cause changes in arterial blood gases [3–5], inducing the “diving response” which includes bradycardia, reduced cardiac output, increased arterial blood pressure, and peripheral vasoconstriction [6]. This complex adaptation mechanism is caused by the simultaneous activation of the sympathetic and parasympathetic nervous system that seems to reduce O_2_ consumption in peripheral tissues to ensure enough O_2_ supply of the vital organs [7]. Blood is rerouted to brain, heart [8, 9], liver [10], and the active muscles [11] as a result of the diving response to optimize O_2_ management and help prolonging apnea duration [12, 13]. BH-diving carries several risks related to physiological stressors that arise in the extreme environment as a consequence of the increased hydrostatic pressure, hypercapnia, hypothermia, and strenuous exercise [4].

A prolonged strenuous physical activity may result, also in not extreme environments, in an increased exercise-induced muscle fatigue, in both skeletal and cardiac muscles [14] that can induce an increase of muscles injury markers such as creatine kinase (CK), aspartate transferase (AST), and alanine transferase (ALT) when concerning the skeletal muscle and cardiac creatine kinase isoenzyme (CK-MBm), and cardiac troponin I (cTnI) when concerning the cardiac muscle [15, 16]. Lactate dehydrogenase (LDH) could also be used as a marker of muscle stress [17].

These enzymes are commonly studied as stress markers of specific organs or tissue (i.e., muscle) where they are more represented.

CK is an enzyme expressed by various tissues, especially those that need high amounts of ATP such as skeletal muscle, brain, heart [18], where it catalyzes the reversible conversion of creatine and uses adenosine triphosphate (ATP) to create phosphocreatine (PCr) and adenosine diphosphate (ADP). CK is also involved in smooth muscle energetic system but with relatively low concentrations as compared to that of skeletal muscle [19]. CK is also considered a qualitative marker for skeletal muscle microtrauma [20]: its changes during physical activity depend on individual physiology, training level, exercise duration, with peak values recorded after endurance events [21].

Aspartate transferase (AST) and alanine transferase (ALT) are commonly considered liver damage markers [22]. ALT is found mainly in the liver but also, in smaller amounts, in the kidney, heart, muscle, and pancreas while AST is present in the liver as well as in considerable amounts in other tissues including muscles [23]. AST and ALT are released from activated muscles, and levels can increase after acute physical exercise: increase in AST and ALT activity is related to the type, intensity, and duration of physical effort [24]. The increase in AST activity during intense exercise is more likely connected to the release of the enzyme from the muscle cells than to liver pathologies [25–28].

CK-MBm is the CK isoenzyme present in cardiac muscle. Similar to CK, the influence of physical exercise on CK-MBm is related to the type, intensity, and duration of muscular activity. The elevation of serum CK-MBm is not unique to athletes but is a common occurrence among individuals who perform eccentric exercise [29, 30]. Prolonged endurance exercise may be associated with increase in serum CK and CK-MBm levels that are comparable to those of a myocardial infarction [31].

A cardiac biomarker typically seen with myocardial infarction is cardiac troponin I (cTnI) [15, 16]. Troponins (T and I) are commonly used diagnostic markers for cardiac ischemia and infarction. Increased circulating troponins can be related to strenuous physical exercise such as marathon competition [32].

In skeletal and smooth muscle, LDH catalyzes the interconversion of pyruvate and lactate with concomitant interconversion of NADH and NAD^+^ to meet the energy demand of the cell. A closer, mechanistic analysis of lactate production under anaerobic conditions shows that there is no biochemical evidence for the production of lactate through LDH contributing to acidosis. While LDH activity is correlated to muscle fatigue, the production of lactate by means of the LDH complex works as a system to delay the onset of muscle fatigue [33].

Despite all the available information in normobaric condition, very few data are known about cardiac and skeletal muscle serum markers in BH-diving.

In particular, there are not information regarding if the possible variations of mentioned biomarkers could be related to the physical effort and the hypoxia or a combination of both. We hypothesized that the combined environmental stress factors (cold, hyperbaric pressure exposure) associated with physical activity during BH-diving would be more predominant compared to the hypoxia.

The aim of this study is to evaluate serum cardiac and skeletal muscle stress markers in elite BH-divers after a BH-diving training session.

## Material and Methods

### Subjects and Diving Protocol

A total of 12 expert healthy BH-divers, 9 males and 3 females, were investigated during an open sea training session at Elba island, Italy. All the divers were informed about risks and benefits of this study, read and signed a specific informed consent form before the experiment and provided personal anthropometric parameters. The study was conducted in accordance with the Helsinki Declaration.

The study was conducted in accordance with the Helsinki Declaration and was approved by the Ethical Committee of the Università degli Studi di Milano, Italy (Aut. No. 37/17).

The selected volunteers are labeled “expert” because they are affiliated to the “Apnea Academy” Training Agency as instructors or high-level BH-divers, and are able to reach a minimum of 30 m in constant weight, 4 min static apnea (at the surface), 75 m dynamic BH-diving (horizontal) in a swimming pool (distance).

The exclusion criteria were as follows: history or clinical evidence of hypertension, cardiac, pulmonary, or any other significant disease; any acute illness during the 15 days before the experiment; use of aspirin, paracetamol, or other anti-inflammatory drugs in the 7 days before the experiment; compressed-gas diving during the 30 days before the test.

As per Apnea Academy standard procedures, all the divers performed their usual training with a freely determined number and time of warm-up dives, bottom time, and surface intervals, after this all the subjects gradually approached the maximum daily personal depth (with a free number of dives) and, when ready, performed the last dive reaching the maximum depth of the training session.

Diving profiles—including mean depth, maximum depth, and number of dives—were recorded using a free-diving computer (UP-X1 Omersub Spa, Monza Brianza, Italy).

This computer measured and recorded diving data every 2 s and allowed for the calculation of the maximum gradient factor (GF) according to the Buhlmann ZHL16 C model.

### Blood Draw Protocol

A butterfly needle (21G × 34 0.8 × 19 mm Green) was placed in the antecubital vein to collect 5 ml of blood using 5 ml serum containing tube (Vacutainer, Becton, Dickinson and Company, Franklin Lakes, NJ, USA).

We collected blood samples per each of the following time steps:

- Basal: 30 min before the start of the warm-up
- T0: 30 min after the dive sessions
- T1: 4 h after the dive session

After 15 min and before 30 min from collection at room temperature, blood samples were centrifuged (3000 rpm for 10 min) to separate serum from cell fraction and was frozen at −20 °C. Then, the serum samples were then delivered to the laboratory for analysis and kept at −20 °C until the analysis.

Creatine kinase (CK), aspartate transaminase (AST), alanine aminotransferase (ALT), creatine kinase isoenzyme (CK-MBm), cardiac troponin I (cTnI), and lactate dehydrogenase (LDH) were measured in serum samples.

Serum samples were defreezed, at room temperature shacked with the Vortex for 5 s for homogenization before the analysis. Briefly, 500 μl of each sample were placed in a plastic test tube. The test tubes were allocated in the auto sampler of a VITROS® 5600 (Ortho-Clinical Diagnostics, High Wycombe, United Kingdom). The reported total imprecision was < 2.8 %, while the intra assay CV % was < 1.8%. The reference value of the laboratory tests is the following: CK 30-135 U/L, AST 17-59 U/L, ALT <50 U/L, CK-MBm < 6.73 ng/mL (male), and < 3.77 ng/mL (female), LDH 120-146 U/L.

### Statistical Analysis

Data are presented as mean±standard deviation (SD) for parametric data and median, or range for non-parametric data. To minimize the subject-to-subject variability, data are normalized against the basal value. The D’Agostino and Pearson normality test was used to assume a Gaussian distribution. Then, data were analyzed by either the one-way ANOVA for multiple comparison, or the Friedman test for multiple comparison, parametric or non-parametric data respectively. A probability lower than 5% was assumed as the threshold to reject the null hypothesis (*p* < 0.05) and 11 degrees of freedom for all the test (95% of confidence interval).

The datasets generated and analyzed during the current study are available from the corresponding author upon request.

## Results

A total of 12 experienced breath-hold divers, 9 male and 3 female, mean age 41.6 ± 5.6 years, mean height 178.6 ± 9.8 cm; mean weight 76.5 ± 12.8 kg and BMI 23.8 ± 2.2 were investigated (Additional file 1: Table S1).

The diving profile showed a mean of maximum depth of 33.3 meters +/− 6.3, a mean of numbers of dives of 14.8 +/− 3.2 and a mean of average depth of 18.9 +/− 4.6 (Additional file 1: Table S1). All the subjects respected the warm-up protocol. All the volunteers completed the experiment without Taravana episodes, evidence of pulmonary, and/or ear barotraumas or other health problems.

Diving was performed at Elba island, Italy, in salt water at 21 ± 0.5 °C mean temperature, as recorded by diving computer.

**Fig. 1:**
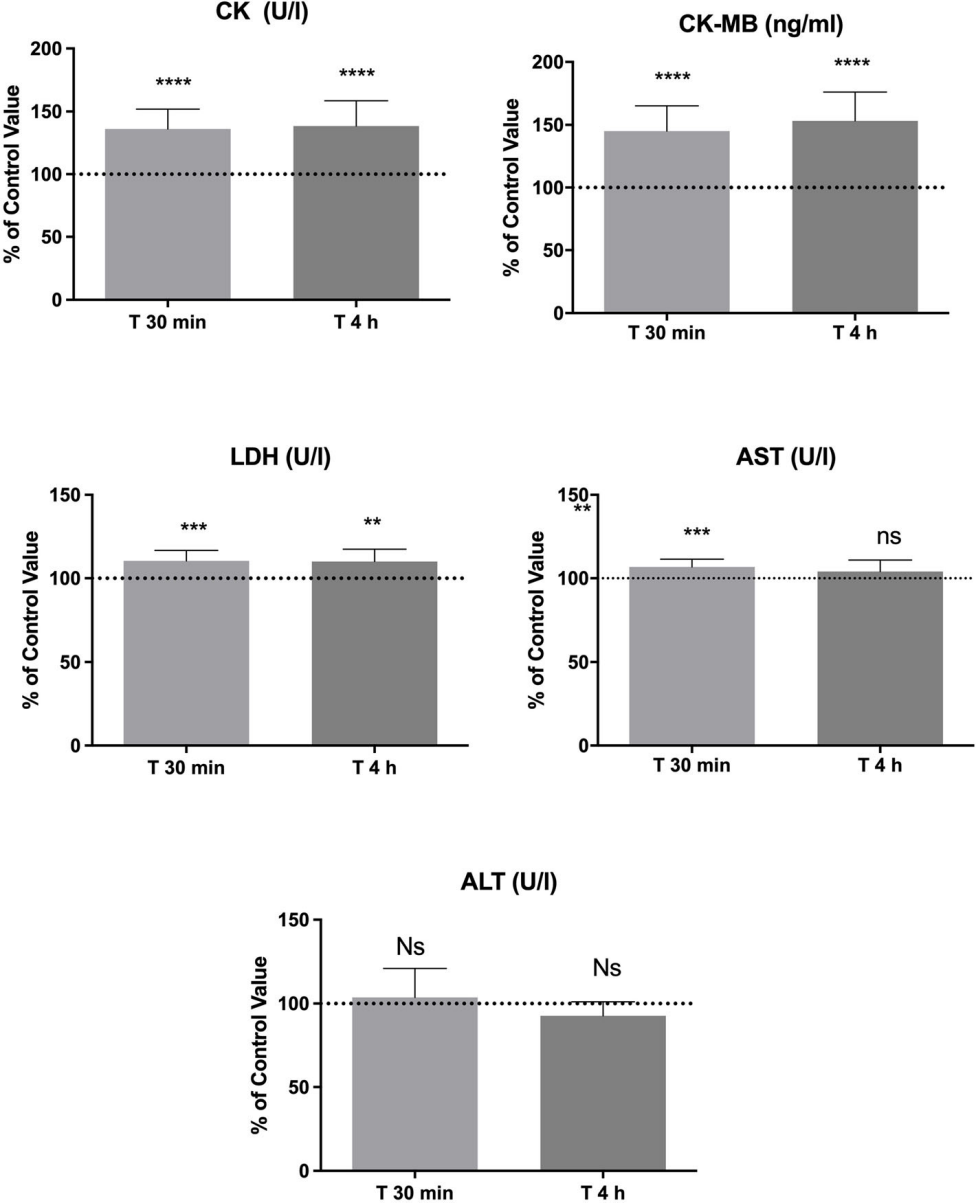
Serum levels of cardiac and skeletal muscle stress biomarker changes after a session of BH-diving training. Data are presented as mean ± standard deviation (SD). The height of the columns represents means and the error bars represent sd’s. *p < 0.05, **p < 0.01, ***p < 0.001, ****p < 0.0001

As reported in Fig. 1, we found statistically significant increases of CK (T0: 136.1% ± 15.8 *p* < 0.0001; T1: 138.5% ± 20.1, *p* < 0.0001), CK-MBm (T0: 145.1% ± 20.1, *p* < 0.0001; T1: 153.2% ± 23.1, *p* < 0.0001) LDH (T0: 110.4% ± 6.4, *p* = 0.0003; T1: 110.1% ± 7.4, *p* = 0.0013) in both T0 and T1 blood samples, as compared to basal value. AST showed a statistically significant increase only at T0 (106.8% ± 4.6, *p* = 0.0007) while ALT did not show any statistical difference in T0 and T1. None of subject show an increase of cTnI levels in post diving samples, in this marker all subjects show the same results before and after the BH-diving session (< 0.012 ng/mL) and the statistical analysis was not applicable.

## Discussion

This study aimed at investigating certain cardiac and skeletal muscle stress marker changes related to BH-diving in elite BH-divers after an open sea training session.

The 12 subjects, 9 males and 3 females, gave consent to the protocol and the blood sampling at the noted times: all were an elite group of “experienced” free divers performing similar exposures regularly: their body characteristics distinguish their excellent physical status.

Increase of CK may be aimed at protecting tissues from hypoxia, as demonstrated by some authors [34]. On the other hand, increased CK can be considered a consequence of increased anaerobic respiration stimulated by muscular activity and pressure-induced peripheral hypoxia [35]. The CK raise might confirm the main role of “diving response” for its intervention in the regulation of hypoxia-inducible factor (HIF-1) expression and activity. CK can be also related to a hyperactivity of breath muscles and hypoxic stress induced by BH-diving [36]. During mild hypoxia, CK probably raises for energetic needs because physical activity requires energy substrates to support aerobic glycolysis. CK is used to sustain ATP production, a condition related to all the kinases involved in energy production [37].

We observed a small elevation in serum AST, however, far from levels indicating liver or skeletal or cardiac muscle injuries [38]. The increase in AST levels is a consequence of a strenuous exercise, which can cause an aminotransferase raise in the absence of liver injury [39]. AST can be used as markers of oxygen desaturation: In hypoxia conditions, Norman et al. found a strong correlation between hypoxia and aminotransferase levels [40]. Since the BH-divers did not perform an intense smooth muscle activity, AST increase may be only due to the myocyte oxygenation. These changes are not confirmed by ALT that on the contrary did not show any change BH-diving related.

CK-MBm isoenzyme, located in cardiac muscle, may be increased due to the stretching of cardiac atria, induced by the increased hydrostatic pressure induced blood shift [41].

The greater CK-MBm concentration facilitates production of ATP from creatine phosphate for muscle contraction, suggesting that the increased CK-MBm may be due to the muscle disruption as consequence of the prolonged physical activity [42, 43]. After exercise, elevated CK-MBm values seem to be associated with the regeneration of new muscle fibers from muscle fiber necrosis caused by exercise [31, 44–46].

cTnI level are unchanged after breath-hold diving training session. Some authors described troponin changes related to electrographic abnormalities or stroke volume decrease [47–49], observed in BH-divers and attributed this to the effect of pressure and/or hypoxemia. However, cTnI level can be also related to the BH-diving technique: Marlinge et al. showed an increase in cTnI during apnea session, probably a consequence of prolonged cardiac workload and hypoxemia [50]. However, we did not observe these variations: a possible explanation may be due to the individual physiology, similar to a training-induced adaptation. Other factors may be dive activity years, personal depth record, number of weekly training sessions, and different BH-diving techniques.

LDH increased after the BH-diving training session, as a consequence of prolonged physical activity. Lactate degradation is fast: a persistence of LDH value higher than pre diving condition may be related to the experience and training of the divers. In hypoxic conditions, the CK raise may be due to energy request to sustain aerobic glycolysis: the creatine phosphate cleavage supports the energy production from ATP. Any similar condition is related by to an increase of the kinase enzymes involved in energy production [37]. In BH-divers, it is possible that the combination of physical activity with hypoxia could raise the CK level.

Our data seem to indicate that during a BH-diving training session, skeletal and cardiac muscles react to physical effort releasing stress-related substances, similar to those observed during intense sports activities (endurance). Although our data agree with those observed in other sports activities, the peculiar nature of BH-diving makes it difficult to understand if our results are related only to exercise-induced muscle adaptation or whether acute hypoxia or a response to environmental changes (pressure) play a role to explain the observed changes, especially considering the additional difficulty in defining the role of hypoxia that recent observations showed occurring only in the final part of BH-diving (ascent phase) [51]. In this regard, it is intriguing to note that these muscle adaptations, comparable with those occurring in endurance sports, are difficult to justify in BH-diving where significant muscle activity occurs only for a limited time, as body movements are effective but not explosive in order to optimize oxygen consumption, and the majority of a BH-diving training session is spent for surface recovery while the underwater time is shorter. Possibly, this BH-diving muscle response could be also explained, besides cardiac and skeletal muscle work, by the particular muscle activity conditions (increase of pressure, hypoxia, diving response) requiring several adaptations mechanisms including smooth muscle-mediated massive vascular response.

It could also be possible that the effects on smooth muscle activity, negligible in normobaric conditions, play a more significant role in BH-diving due to the blood shift-related continuous blood flow regulation.

Further tests will be necessary to better understand the origin of these serum biomarkers changes, focusing separately on the different BH-diving protocols and related risk factors (muscle work, increase of pressure, hypoxia, diving response).

### Limitation

The main limitation of this study is the reduced sample size in this study and that we evaluated only these serum biomarkers in subjects performing constant weight apnea. Could be interesting in the future comparing the results found also in volunteers performing static and dynamic apnea.

## Conclusion

BH diving is associated to prolonged physical activity that can lead to a change in some serum cardiac and muscle stress markers. Post dive data showed an increase of CK, AST, ALT, LDH, and CK-MBm probably due to physical exercise, but other factors such as increased ambient pressure, hypoxia, and diving response could also play a role. No cTnI changes were observed.

## Supplementary Information

**Additional file 1: Table S1.** Typology and characteristics of study groups.

## Data Availability

The datasets generated during and/or analyzed during the current study are available from the corresponding author.

## Abbreviations

ALT: Alanine transferase
AST: Aspartate transferase
BH-diver: Breath-hold diver
BH-diving: Breath-hold diving
BMI: Body mass index
CK: Creatine kinase
CK-MBm: Cardiac creatine kinase isoenzyme
cTnI: Cardiac troponin I
LDH: Lactate dehydrogenase

## References

[1] Edmonds, C.L., C; Pennefather, J. History of diving. Reprinted from ‘Diving and Subaquatic Medicine’ (1975). 1975; Available from: http://archive.rubicon-foundation.org/5894.

[2] Ashcroft, F.M., Life at the extremes, ed. H. Collins 2001, London.

[3] Lindholm P, Lundgren CE (2009). The physiology and pathophysiology of human breath-hold diving. J Appl Physiol (1985).

[4] Pendergast DR, Moon RE, Krasney JJ, Held HE, Zamparo P (2015). Human physiology in an aquatic environment. Compr Physiol.

[5] Bosco G, Rizzato A, Martani L, Schiavo S, Talamonti E, Garetto G, Paganini M, Camporesi EM, Moon RE (2018). Arterial blood gas analysis in breath-hold divers at depth. Front Physiol.

[6] Heusser K, Dzamonja G, Tank J, Palada I, Valic Z, Bakovic D, Obad A, Ivancev V, Breskovic T, Diedrich Á, Joyner MJ, Luft FC, Jordan J, Dujic Z (2009). Cardiovascular regulation during apnea in elite divers. Hypertension.

[7] Dujic Z, Breskovic T, Ljubkovic M (2011). Breath hold diving: in vivo model of the brain survival response in man?. Med Hypotheses.

[8] Andersen HT (1966). Physiological adaptations in diving vertebrates. Physiol Rev.

[9] Butler PJ, Woakes AJ (1987). Heart rate in humans during underwater swimming with and without breath-hold. Respir Physiol.

[10] Kyhl K, Drvis I, Barak O, Mijacika T, Engstrøm T, Secher NH, Dujic Z, Buca A, Madsen PL (2016). Organ perfusion during voluntary pulmonary hyperinflation; a magnetic resonance imaging study. Am J Physiol Heart Circ Physiol.

[11] Gooden BA (1994). Mechanism of the human diving response. Integr Physiol Behav Sci.

[12] Andersson J, Schagatay E (1998). Arterial oxygen desaturation during apnea in humans. Undersea Hyperb Med.

[13] Schagatay E, Andersson J (1998). Diving response and apneic time in humans. Undersea Hyperb Med.

[14] Shave R (2004). Altered cardiac function and minimal cardiac damage during prolonged exercise. Med Sci Sports Exerc.

[15] Leischik R, Spelsberg N (2014). Endurance sport and “cardiac injury”: a prospective study of recreational ironman athletes. Int J Environ Res Public Health.

[16] Tulloh L, Robinson D, Patel A, Ware A, Prendergast C, Sullivan D, Pressley L (2006). Raised troponin T and echocardiographic abnormalities after prolonged strenuous exercise--the Australian Ironman Triathlon. Br J Sports Med.

[17] Kemp M, Donovan J, Higham H, Hooper J (2004). Biochemical markers of myocardial injury. Br J Anaesth.

[18] Wallimann T, Wyss M, Brdiczka D, Nicolay K, Eppenberger HM (1992). Intracellular compartmentation, structure and function of creatine kinase isoenzymes in tissues with high and fluctuating energy demands: the ‘phosphocreatine circuit’ for cellular energy homeostasis. Biochem J.

[19] Clark JF (1994). The creatine kinase system in smooth muscle. Mol Cell Biochem.

[20] Clarkson PM, Hubal MJ (2002). Exercise-induced muscle damage in humans. Am J Phys Med Rehabil.

[21] Kratz A, Lewandrowski KB, Siegel AJ, Chun KY, Flood JG, van Cott EM, Lee-Lewandrowski E (2002). Effect of marathon running on hematologic and biochemical laboratory parameters, including cardiac markers. Am J Clin Pathol.

[22] Burger-Mendonca M, Bielavsky M, Barbosa FC (2008). Liver overload in Brazilian triathletes after half-ironman competition is related muscle fatigue. Ann Hepatol.

[23] Xing-Jiu Huang X, Choi Y, Im H, Yarigama O, Yoon E, Kim H (2006). Aspartate aminotransferase (AST/GOT) and alanine aminotransferase (ALT/GPT) detection techniques Sensors.

[24] Nagel D, Seiler D, Franz H, Jung K (1990). Ultra-long-distance running and the liver. Int J Sports Med.

[25] Banfi G, Morelli P (2008). Relation between body mass index and serum aminotransferases concentrations in professional athletes. J Sports Med Phys Fitness.

[26] Cazzola R, Russo-Volpe S, Cervato G, Cestaro B (2003). Biochemical assessments of oxidative stress, erythrocyte membrane fluidity and antioxidant status in professional soccer players and sedentary controls. Eur J Clin Invest.

[27] Gravina L, Ruiz F, Lekue JA, Irazusta J, Gil SM (2011). Metabolic impact of a soccer match on female players. J Sports Sci.

[28] Lippi G, Schena F, Montagnana M, Salvagno GL, Banfi G, Guidi GC (2011). Significant variation of traditional markers of liver injury after a half-marathon run. Eur J Intern Med.

[29] Buckley-Bleiler R (1989). Serum creatine kinase activity after isometric exercise in premenopausal and postmenopausal women. Exp Aging Res.

[30] Friden J, Sjostrom M, Ekblom B (1983). Myofibrillar damage following intense eccentric exercise in man. Int J Sports Med.

[31] Apple FS, Rogers MA, Sherman WM, Ivy JL (1984). Comparison of serum creatine kinase and creatine kinase MB activities post marathon race versus post myocardial infarction. Clin Chim Acta.

[32] Regwan S (2010). Marathon running as a cause of troponin elevation: a systematic review and meta-analysis. J Interv Cardiol.

[33] Tesch P, Sjödin B, Thorstensson A, Karlsson J (1978). Muscle fatigue and its relation to lactate accumulation and LDH activity in man. Acta Physiol Scand.

[34] Zervou S, Whittington HJ, Ostrowski PJ, Cao F, Tyler J, Lake HA, Neubauer S, Lygate CA (2017). Increasing creatine kinase activity protects against hypoxia/reoxygenation injury but not against anthracycline toxicity in vitro. PLoS One.

[35] Bark DH, Smith LS (1982). Creatine phosphokinase activities in rainbow trout, Salmo gairdneri, associated with rapid decompression. Enzyme.

[36] Lentini S, Manka R, Scholtyssek S, Stoffel-Wagner B, Lüderitz B, Tasci S (2006). Creatine phosphokinase elevation in obstructive sleep apnea syndrome: an unknown association?. Chest.

[37] Egan B, Zierath JR (2013). Exercise metabolism and the molecular regulation of skeletal muscle adaptation. Cell Metab.

[38] Lee TH, Kim WR, Poterucha JJ (2012). Evaluation of elevated liver enzymes. Clin Liver Dis.

[39] Pavletic AJ, Pao M (2015). Exercise-induced elevation of liver enzymes in a healthy female research volunteer. Psychosomatics.

[40] Norman D, Bardwell WA, Arosemena F, Nelesen R, Mills PJ, Loredo JS, Lavine JE, Dimsdale JE (2008). Serum aminotransferase levels are associated with markers of hypoxia in patients with obstructive sleep apnea. Sleep.

[41] Theunissen S, Guerrero F, Sponsiello N, Cialoni D, Pieri M, Germonpré P, Obeid G, Tillmans F, Papadopoulou V, Hemelryck W, Marroni A, de Bels D, Balestra C (2013). Nitric oxide-related endothelial changes in breath-hold and scuba divers. Undersea Hyperb Med.

[42] Baird MF (2012). Creatine-kinase- and exercise-related muscle damage implications for muscle performance and recovery. J Nutr Metab.

[43] Siegel AJ, Silverman LM, Evans WJ (1983). Elevated skeletal muscle creatine kinase MB isoenzyme levels in marathon runners. JAMA.

[44] Apple FS, Rogers MA, Sherman WM, Costill DL, Hagerman FC, Ivy JL (1984). Profile of creatine kinase isoenzymes in skeletal muscles of marathon runners. Clin Chem.

[45] Apple FS (1985). Creatine kinase-MB isoenzyme adaptations in stressed human skeletal muscle of marathon runners. J Appl Physiol.

[46] Rogers MA, Stull GA, Apple FS (1985). Creatine kinase isoenzyme activities in men and women following a marathon race. Med Sci Sports Exerc.

[47] Marongiu E, Crisafulli A, Ghiani G, Olla S, Roberto S, Pinna M, Pusceddu M, Palazzolo G, Sanna I, Concu A, Tocco F (2015). Cardiovascular responses during free-diving in the sea. Int J Sports Med.

[48] Zelenkova I, Chomahidze P (2016). Long-term effects of frequent maximal breath-holding on the cardiac health of elite freedivers. Scand J Med Sci Sports.

[49] Gargne O, Joulia F, Golé Y, Coulange M, Bessereau J, Fontanari P, Desruelle AV, Gavarry O, Boussuges A (2012). Cardiac alterations induced by a fish-catching diving competition. Scand J Med Sci Sports.

[50] Marlinge M, Coulange M, Fitzpatrick RC, Delacroix R, Gabarre A, Lainé N, Cautela J, Louge P, Boussuges A, Rostain JC, Guieu R, Joulia FC (2019). Physiological stress markers during breath-hold diving and SCUBA diving. Physiol Rep.

[51] Bosco G, Rizzato A, Moon RE, Camporesi EM (2018). Environmental physiology and diving medicine. Front Psychol.

