# Supplementary material for: Serum Cardiac and Skeletal Muscle Marker Changes in Repetitive Breath-hold Diving
Source: Sports Med Open. 2021 Aug 21;7:58. doi: 10.1186/s40798-021-00349-z (PMC8380208; doi:10.1186/s40798-021-00349-z)
Supplement: Supplementary file 1 — Additional file 1: Table S1. Typology and characteristics of study groups. [file 40798_2021_349_MOESM1_ESM.docx]

Table S1. Typology and characteristics of study groups

A) Anthropometric data;

B) Diving profile

A)

|  | Antropometric data | | | | |
| --- | --- | --- | --- | --- | --- |
| **ID** | **Gender** | **Age** | **Height** | **Weight** | **BMI** |
| 1 | M | 38 | 1,80 | 82 | 25,3 |
| 2 | M | 42 | 1,82 | 78 | 23,5 |
| 3 | M | 35 | 1,80 | 84 | 25,9 |
| 4 | F | 30 | 1,70 | 57 | 19,7 |
| 5 | F | 42 | 1,78 | 68 | 21,5 |
| 6 | F | 44 | 1,55 | 54 | 22,5 |
| 7 | M | 39 | 1,82 | 77 | 23,2 |
| 8 | M | 49 | 1,88 | 98 | 27,7 |
| 9 | M | 44 | 1,86 | 76 | 22,0 |
| 10 | M | 48 | 1,68 | 69 | 24,4 |
| 11 | M | 48 | 1,89 | 87 | 24,4 |
| 12 | M | 40 | 1,85 | 88 | 25,7 |

| **Sample description**  **summary (N=12)** |  |  |
| --- | --- | --- |
| Gender | Female=3 (25%) | Male=9 (75%) |
| AGE | 41.6 years | +/- 5.6 |
| Height | 178.6 cm | +/- 9.8 |
| Weight | 76.5 kg | +/- 12.8 |
| BMI | 23.8 | +/- 2.2 |

B)

|  | Diving Profile | | |  |
| --- | --- | --- | --- | --- |
| **ID** | **Max Depth** | 16 | **Mean Depth** |  |
| 1 | 28,7 | 17,2 | 16 |  |
| 2 | 30,8 | 27,3 | 17,2 |  |
| 3 | 31,9 | 18 | 27,3 |  |
| 4 | 39,6 | 22,5 | 18 |  |
| 5 | 40,3 | 11,5 | 22,5 |  |
| 6 | 25 | 22,3 | 11,5 |  |
| 7 | 35 | 20,2 | 22,3 |  |
| 8 | 32 | 20,2 | 20,2 |  |
| 9 | 35 | 13 | 20,2 |  |
| 10 | 28 | 23,7 | 13 |  |
| 11 | 46,5 | 15 | 23,7 |  |
| 12 | 27 | 11 | 15 |  |

| **Diving Profile Summary** |  |  |
| --- | --- | --- |
| Maximum depth | 33.3 Meters | +/- 6.3 |
| N° of dives | 14.8 | +/- 3.2 |
| Mean of depth | 18.9 | +/- 4.6 |
